# Serum Levels of IL-22 in Patients with Oral Lichen Planus and Cutaneous Lichen Planus

**DOI:** 10.30476/DENTJODS.2020.84667.1096

**Published:** 2020-12

**Authors:** Maryam Mardani, Shiva Torabi Ardakani, Ladan Dastgheib, Nasrin Hamidizadeh

**Affiliations:** 1 Oral and Dental Disease Research Center, School of Dentistry, Shiraz University of Medical Sciences, Shiraz, Iran; 2 Molecular Dermatology Research Center, Shiraz University of Medical Sciences, Shiraz, Iran

**Keywords:** IL-22, Oral lichen planus, Cutaneous lichen planus, Serum

## Abstract

**Statement of the Problem:**

Lichen planus disease is a chronic inflammatory disorder of mucosal and cutaneous tissues with yet unclear etiology and pathogenesis. Cytokines play an important role in the initiation, maintenance of inflammatory and intercellular crosstalk.

**Purpose:**

We assessed serum levels of IL-22 in patients with oral and cutaneous lichen planus and made comparison with healthy individuals.

**Materials and Method:**

In this case-control study, peripheral blood samples of 40 patients with lichen planus disease, included two groups of oral lichen planus (n=20) and cutaneous lichen planus (n=20) were compared with 32 healthy individuals in this case-control study. Serum samples were prepared from patients with lichen planus and IL-22 concentration was measured in each serum sample by using a commercial ELISA Kit. The obtained data were then analyzed using the Kruskal-Wallis (one-way ANOVA) test.

**Results:**

The IL-22 serum levels were significantly higher in patients with oral lichen planus compared to the healthy control group (*p*< 0.001). No statistically significant differences were observed in serum levels of IL-22 in cutaneous lichen planus patients compared to the controls (*p*= 0.183).

**Conclusion:**

Increased IL-22 serum levels in patients with oral lichen planus may play an important role in the pathogenesis of oral lichen planus. The administration of the recombinant or antagonist of IL-22 could be a new therapeutic opportunity in the treatment of oral lichen planus.

## Introduction

Lichen planus (LP) is a chronic and T cell-mediated inflammatory disorder of the skin and mucous membranes, which is classified based on the tissue involved, such as oral lichen planus (OLP), cutaneous lichen planus (CLP) and oral-cutaneous lichen planus [ 1 - 2 ]. 

LP affects about 0.5-2% of the general population, especially females, and most often occurs in middle age [ 3 ]. LP lesions can be symptomless, annular, linear, hypertrophic, atrophic, bullous, ulcerative, and pigmented [ 4 ]. Clinically, CLP is characterized by purple, polygonal, pruritic papules frequently covered by Wickham striae, located on the flexor surface, of the wrist, the shins, the trunk, and the medial thighs [ 5 ]. A basic clinical diagnosis of OLP can be made by Wickham striae [ 6 ].

It is reported that 0.4% to 5.3% of OLP lesions are transformed into carcinoma, making it a pre-malignant condition [ 7 ]. The etiology and pathogenesis of LP is unclear, but the combination of mast cell degranulation and matrix and matrix metalloproteinase activation in OLP lesions leads to T cell accumulation in the superficial lamina propria, basal membrane disruption, intraepithelial T cell migration, and keratinocyte apoptosis [ 8 ]. The majority of infiltering immune cells into epithelial layer and lamina propria are CD8+T and CD4+T cells, respectively [ 8 - 9 ]. Local and systemic inducers of cell-mediated hypersensitivity, autoimmune responses to epithelial antigens, viral infections, some systemic medications, dental material, stress, genetics, and tobacco chewing have been reported to be the triggering factors for OLP [ 10 - 11 ].

Cytokines play an important role in recruitment, differentiation, and activation of immune cells into inflammatory sites. Cytokines are soluble proteins with a low molecular weight that are produced by various cells, which play a complex role in the initiation, progression, and regression of autoimmune inflammation [ 12 ]. They regulate the immune responses by binding to cell surface receptors. IL-22 is a member of IL-10 superfamily that uses IL-22R1/IL-10R2 for cell signaling in target cells, produced by Th17, Th22, NK, innate lymphoid cells and various types of CD4+ and CD8+ T lymphocytes [ 13 ]. The IL-22 production is expressed in IL-23 in a dependent manner [ 14 - 16 ]. Several studies showed that IL-23, IL-18, and IL-6 increase the release of IL-22 [ 17 - 18 ]. IL-22 plays an important role in immune responses, par ticularly in inflammatory and autoimmune diseases in humans and animals [ 19 ]. IL-22 might have a protective, regenerative, or pathogenic role depending on the disease stage [ 20 - 22 ]. 

It is reported that IL-22 is involved in the development and pathogenesis of several autoimmune diseases such as systemic lupus erythematosus, rheumatoid arthritis, multiple sclerosis, sjögren syndrome, and psoriasis [ 21 ]. IL-22 has a protective role in liver diseases and graft versus host disease [ 23 ], an anti-apoptotic role in rheumatoid arthritis [ 24 ] and an anti-inflammatory role in asthma [ 25 ]. Moreover, IL-22 induces psoriasis-like lesions in mice [ 15 ].

Furthermore, IL-22 might be involved in the defense against viruses [ 26 - 27 ], candida albicans [ 28 ] and bact-eria [ 16 , 29 - 31 ]. Several studies have investigated the expression of cytokines, such as TNF- α, IFN-γ, TGF-β, IL-1, 2, 4, 5, 6, 8, 10, 12, 17, 18, and IL-22 in serum, saliva, and lesions in OLP patients [ 32 - 36 ].

Several authors [ 34 - 36 ] have suggested that different cytokines play a role in the initiation and progression of oral lichen planus. Hence, identifying and introducing appropriate immunological targets for diagnostic and therapeutic usage is essential. IL-22 plays a key role in mucosal barrier defense, tissue repair, survival, and proliferation of epithelial cells, but evidence demonstrated both the protective and the pathogenic properties of IL-22 in autoimmune disease, infection, and malignancy [ 37 ]. In the current study, we scrutinized if the IL-22 serum level was different in LP in different forms such as OLP and CLP. Therefore, we measured IL-22 levels in patients’ sera with OLP and CLP in comparison with healthy controls.

## Materials and Method

In this study, 20 OLP patients and 20 CLP patients were selected from individuals, who referred to the dental school, and Molecular Dermatology Research Center, Shiraz University of Medical Sciences, Shiraz, Iran (Table 1). Clinical diagnosis and classification of OLP were done by an oral and maxillofacial specialist, and clinical diagnosis of CLP was performed by a dermatologist and confirmed by a pathologist. The IL-22 concentrations in patients and healthy controls were measured using enzyme-linked immunosorbent assay (ELISA) kit (My Biosource, San Diego, California, USA). Results were expressed in pg/ml for IL-22 in serum.

**Table 1:** Baseline characteristics of the investigated patients with oral lichen planus, cutaneous lichen planus, and controls

Diagnosis	N	Mean±Std(age)	Age range	Women (n)	Men (n)
Oral LP	20	51.31±13.68	28 ~ 84	16	4
Cutaneous LP	20	43.53±15.95	17 ~72	14	6
Control	32	48.68±9.95	17 ~ 65	25	7

Furthermore, participants were asked if they smoked cigarette, use any drugs, had a history of any surgery, infectious diseases, vaccination, trauma, immune therapy, and chemotherapy. All individuals with items listed above and those with a history of allergy, autoimmunity, immunodeficiency, and cancer were excluded. The control group included 32 healthy individuals who were selected from healthy blood donors in the same age range and gender. Informed consent was obtained from all participants before blood donation and data publication. This study was approved by the local Ethics Committee of Shiraz University of Medical Science (EC-SUMS).

### Sampling

Approximately 5 ml of peripheral blood samples were collected from each participant, and the sera were separated in 3500 rpm for 10 min, stored at -20ºC until further usage.

### Statistical analysis

Data were analyzed using the SPSS software (version 16, Chicago, IL, USA). Kolmogorov-Smirnov and Sha-piro-Wilk tests were used to assess the normal distribution of data. Kruskal–Wallis (one-way ANOVA) test was performed to demonstrate the IL-22 level difference between patients and the controls. P-values less than 0.05 were considered statistically significant.

## Results

In this study, 46 patients with different forms of LP, which included 20 patients with OLP, 20 with CLP and 32 healthy individuals, were examined. There was no significant difference in age between patients and healthy controls. The mean serum levels of IL-22 in patients with different forms of LP and healthy controls are shown in Table 2. The mean serum level of IL-22 in patients with OLP was significantly higher than the healthy controls (*p*< 0.001). No statistically significant differences were observed in serum levels of IL-22 in CLP patients compared to the controls (*p*= 0.183).

**Table 2:** Comparison of IL-22 levels in oral and cutaneous lichen planus with the controls

Variable	IL-22 (pg/ml)± STD	N	*p* Value
Oral LP	32.4 ± 10	20	< 0.001
Control	24.7 ± 11.7	32	
Cutaneous LP	25.95 ± 6.6	20	0.183
Control	24.7 ± 11.7	32	

## Discussion

LP is a chronic autoimmune disease that leads to epithelial cell damage through the infiltration of T lymphocytes and the degeneration of basal keratinocytes. Its mechanism is unclear, but a cytokine complex network plays an important role in the exacerbation and continuity in OLP [ 36 ]. Several studies were showed an abnormal concentration of the levels of various cytokines such as IL-1, 2, 4, 5, 6, 8, 10, 12, 17, 18, 22, TGF-β, IFN-γ, and TNF-α in lesions, serum, OLP. Nonetheless, it is known that some interleukins, saliva, and peripheral mononuclear cells in patients with including TNF-α, IFN-γ, IL-1, 2, 4, 5, 6, 8, 10, 12, 17, 18, 22, are involved in the pathogenesis of OLP [ 32 - 33 ].

IL-22 has both protective and pathogenic roles in different diseases and showed inconsistent results in several studies [ 38 - 40 ]. Furthermore, IL-22 is involved in the pathogenesis of several autoimmune diseases such as systemic lupus erythematosus, rheumatoid arthritis, multiple sclerosis, sjögren syndrome, psoriasis [ 21 ], and skin diseases [ 41 ] but has protective, anti-apoptotic and anti-inflammatory effects in graft versus host disease [ 23 ], rheumatoid arthritis [ 24 ], and asthma [ 25 ]. 

Previous studies also reported significantly increased IL-22 levels in oral biopsies from patients with OLP compared with the normal controls [ 42 - 43 ]. Besides, the IL-22 level was increased in the lesions of OLP patients, but its level in the serum was inconsistent [ 33 ]. On the contrary, Shirazian *et al*. [ 44 ] demonstrated an increase in the salivary levels of IL-22 in controls than OLP patients. The present study also showed an elevated level of IL-22 in the serum of patients with OLP but not in CLP patients. The discrepancy between the different results in cytokine levels in OLP in various studies, especially IL-22, can be attributed to the measurement techniques, the genetic background, the polymorphism, the clinical forms, cytokine milieu, gender, and age [ 32 - 33 , 36 - 40 ]. Elevated levels of IL-22 may be due to its protective function against bacterial infections and tissue antigens [ 43 ] or, its increased level may contribute to the pathogenesis of the OLP. But based on some previous studies [ 33 , 43 ], it seems that the pathogenesis role of IL-22 is more prominent.

## Conclusion

IL-22 may play an important role in the pathogenesis of OLP and might serve as a marker for diagnosis of OLP. Therefore, IL-22 antagonists could be a potential therapeutic option. However, extensive researches are needed.
